# Red mark syndrome of trout (*Oncorhynchus mykiss*; Walbaum, 1792): Histopathological scoring and correlation with gross lesions

**DOI:** 10.1111/jfd.13391

**Published:** 2021-05-10

**Authors:** M. Galeotti, G. Sarli, R. Sirri, L. Mandrioli, P. Beraldo, P. Bronzatti, R. Giavenni, M. Orioles, G.E. Magi, D. Volpatti

**Affiliations:** ^1^ Veterinary Pathology Unit DI4A University of Udine Udine Italy; ^2^ Department of Veterinary Medical Sciences Alma Mater Studiorum University of Bologna Bologna Italy; ^3^ Fish farm Veterinary Consultant Udine Italy; ^4^ School of Biosciences and Veterinary Medicine University of Camerino Matelica Italy

**Keywords:** gross lesions, histopathology, PCNA, rainbow trout, RMS, skin

## Abstract

Red mark syndrome (RMS) is a skin disorder affecting rainbow trout (*Oncorhynchus mykiss*). The present work aimed to correlate the gross skin lesions affecting 46 fish sampled from farms surveyed for RMS with their microscopic features, identifying histological parameters that may be suggestive of disease progression. Skin lesions were grossly included in one of three categories (types I, II and III) according to the progressive degree of severity. Histological parameters and anti‐proliferating cell nuclear antigen (PCNA) tissue immunoreactivity were semi‐quantitatively assessed. In the dermis, PCNA‐positive lymphocytes, fibroblasts and endothelial cells were indicative of active phlogosis. A significant increase in PCNA‐immunoreactive lymphocytes, from gross type I to type III cases, was found only in the hypodermis. The histological parameters significantly associated with the gross lesion severity were progressive loss of the epithelium and scales, recruitment of inflammatory cells in the *stratum compactum*, loss of architecture of the *stratum compactum*, perivascular and perineural granulomatous inflammation and increase in lymphocyte infiltration of the muscular layer. In the type II and type III categories, inflammation in the hypodermis and muscle displayed a granulomatous pattern, reinforcing the hypothesis of an immunopathological mechanism. The morphological diagnosis of “deep chronic dermatitis associated to panniculitis and myositis, characterised by lympho‐histiocytic and granulomatous reaction” is suggested.

## 1 INTRODUCTION

Red mark syndrome (RMS) is a skin disease affecting rainbow trout (*Oncorhynchus mykiss* Walbaum, 1792). It first emerged in the United Kingdom (Ferguson et al., 2006; Noguera, 2008; Oidtmann & Noguera, 2008; Verner‐Jeffreys et al., 2006, 2008) and then was reported in Finland (Bruno et al., 2007), Austria and Switzerland (Schimidt‐Posthaus et al., 2009), Italy (Galeotti et al., 2011), and more recently in Turkey (Kubilay et al., 2014), Iran (Sasani et al., 2016), Chile (Sandoval et al., 2016), Slovenia (Galeotti, Ronza, et al., 2017), Denmark (Henriksen & Schmidt, 2017), Korea (Oh et al., 2019) and Bosnia and Herzegovina (Galeotti et al., 2021). European RMS strongly correlates with two similar rainbow trout diseases reported in the United States, namely U.S. strawberry disease (USSD) and U.S. Rush (La Patra et al., 1994; Lloyd et al., 2008; Olson et al., 1985). Furthermore, RMS shows morphological similarities with a warm water form of strawberry disease (*SD*) previously described in Europe (Fleury et al., 1985; St‐Hilaire & Jeffery, 2004). It is currently believed that RMS and USSD are the same disease (Oidtmann et al., 2013). The transmissible nature of the disease has been demonstrated by experimental cohabitation trials (von Gersdorff Jørgensen et al., 2019; Verner‐Jeffreys et al., 2008), and the involvement of a *Midichloria*‐like organism (MLO) (Cafiso et al., 2015; Metselaar et al., 2020) has been hypothesized. Intracytoplasmic microorganisms, resembling Rickettsiales, were observed by transmission electron microscopy (TEM) within macrophages, fibroblasts and erythrocytes in the skin of RMS‐affected rainbow trout in Italy and Slovenia (Galeotti, Manzano, et al., 2017; Galeotti, Ronza, et al., 2017). In a recent study, a strong positive correlation was demonstrated between the presence of MLO and RMS‐affected fish, further supported by immunohistochemistry (Metseelar et al., 2020). Early clinical signs are characterized by pale yellow/grey flat patches with central reddening, mainly on the fish flank, or opaque raised patches, with increased mucus production (Schmidt‐Posthaus et al., 2009; Verner‐Jeffreys et al., 2008). The lesion evolution includes an increase in size, elevation and redness of the wounds. The diameter of the lesions ranges from 5 mm to several centimetres; they appear round or ovoid, elongated, with progressive scale loss. Ulceration is not a common finding (Ferguson et al., 2006; Noguera, 2008; Verner‐Jeffreys et al., 2006, 2008). The histology of the affected skin reveals a mild‐to‐severe dermatitis, with intact epidermis and massive infiltration of inflammatory cells, such as lymphocytes, plasma cells and macrophages (involving the *stratum spongiosum* [SS], the scale pockets [SP] and the *stratum compactum* [SC]). A severe infiltration of the same cells is consistently observed in the hypodermis and underlying muscle (Galeotti, Manzano, et al., 2017; Galeotti, Ronza, et al., 2017; McCarthy et al., 2013; Schmidt‐Posthaus et al., 2009; Verner‐Jeffreys et al., 2008). Most of the investigations described in the literature have aimed to understand the aetiopathogenesis of the disease, whereas relatively few papers have focused on a detailed analysis of its histological features in order to both define a classification of the lesions during their evolution and further understand the pathogenesis of this disease, which is still far from being clear. The present work aimed to describe the development of the lesion from the histological point of view, starting from the early stages to the fully advanced lesions, and to correlate the scoring of skin histological patterns with selected macroscopic features of the lesions.

## 2 MATERIALS AND METHODS

### 2.1 Case study and sampling

The disease was identified and surveyed in five rainbow trout farms (namely A, B, C, D and E) located in Northern Italy, throughout the spring–autumn period from 2011 to 2015, when the water temperature was between 9 and 10°C. The percentage of affected fish in the farms was 10%–15%, and their mean weight was around 500 g. Fish did not show alteration of behaviour or other signs of disease except for the cutaneous gross changes ascribable to RMS; mortality was absent. Forty‐six symptomatic fish were sampled and killed by an overdose of MS‐222 (300 mg/L); the number of individuals collected from each farm was as follows: A = 10, B = 10, C = 10, D = 8 and E = 8. Samples of skin, heart, spleen, kidney, liver, gill, brain and gut were included for the histological analysis. A single skin lesion was collected from each symptomatic fish, and then subjected to gross classification and processed for histology. For those individuals presenting multiple skin lesions, the most severe were selected as representative for this purpose.

### 2.2 Gross classification criteria for RMS skin lesions

To classify the skin lesions of RMS‐affected rainbow trout on the basis of gross appearance, the authors employed grading criteria as described by Galeotti et al. (2013) with some modifications. The included criteria for descriptive methodology were chosen considering a high number of skin lesions observed throughout the last years in Italian, Slovenian and Bosnian rainbow trout farms with full‐blown RMS episodes. The five macroscopic parameters employed in this study to grade RMS skin lesions were as follows: size, colour variation, exudate, visible scales and erosion. Size ranging from few mm to several centimetres in diameter and considering the major diameter for the oval or elongated lesions. Colour variation from pale grey‐whitish to bright red haemorrhagic patches. Presence of exudate: mild (sharp demarcated pale areas with slightly raised scales), moderate (flat or slightly raised whitish patches, covered with clear serous exudate associated with multifocal petechiae) or severe (flat or slightly to moderately raised, shiny patches covered with whitish to yellowish serous fibrinous exudate associated with haemorrhages). Presence or absence of visible scales within lesion. Presence of erosion within lesion defined as greyish, sometimes slightly depressed area, usually centrally located and surrounded by prominent redness.

According to these macroscopic descriptors, the lesions were classified into three categories: type I (mild), type II (moderate) and type III (severe), as shown in Table 1. For the inclusion within a category, the lesion evaluated must possess at least three parameters, as described in Table 1. Sampled trout sometimes showed concomitant multiple lesions, sometimes confluent or belonging to different categories. In this study, from each trout under investigation, only one lesion was examined considering the most severe in order to correlate it with the histological features.

**TABLE 1:** Classification of RMS skin lesion based on the macroscopic parameters

Parameters	Type I	Type II	Type III
Size	Up to 1 cm	1–2 cm	Above 2 cm
Colour	Pale grey, whitish, slight redness	From grey to reddish with petechiae	Marked redness and haemorrhages
Exudate	Mild	Moderate	Moderate or severe
Scale	Usually visible	Mild scale loss	Evident scale loss
Erosion	Absent	Absent or rare	Frequent

### 2.3 Histology

Tissue samples obtained from skin/muscle, brain, gill, liver, heart, spleen, kidney and gut were fixed in 4% neutral buffered formaldehyde at 4°C overnight. After fixation, samples were equilibrated at room temperature, processed by an automatic histoprocessor (TISBE tissue processor, Diapath) and embedded in paraffin (Paraplast Plus, Diapath). Serial 5‐μm sections were obtained by a programmable microtome (Reichert‐Jung 2050) and then stained with haematoxylin and eosin and Van Gieson Weigert trichrome. The specimens were examined with a light microscope (Leica DMRB), and images were acquired with a Nikon system. A single observer (MG) scored the histopathological parameters on skin samples, using semi‐quantitative criteria. For each parameter, the score ranged from 1 to 4, as follows: 1 = absence of the lesion, 2 = mild lesion, 3 = moderate lesion and 4 = severe lesion. The parameters evaluated are shown in Table 2.

**TABLE 2:** Median values and range for each group of the scored histological variables and results of the Spearman correlation test with the three gross types for each variable

Site	Histological variable	Gross skin lesion			*R* Spearman's test*
		Type I	Type II	Type III	
Epidermis	Absence	1(1–2)	2(1–4)	3(1–4)	0.64*
	Exocytosis	1(1–2)	2(1–4)	3(2–3)	0.47*
	Necrosis	1	2(1–4)	2(1–4)	0.45*
Dermo‐epidermal junction	Lymphocyte infiltration	2(1–3)	3(3–4)	4(3–4)	0.53*
	Lichenoid‐like detachment	1	1(1–3)	1(1–3)	0.12
	Necrosis	1	3(1–4)	3(1–4)	0.40*
	Haemorrhage	1	2(1–3)	2(1–4)	0.41*
Dermis—stratum spongiosum	Cellular infiltration	2(2–3)	3(3–4)	4(3–4)	0.48*
	Vessel congestion	1	3(2–4)	4(3–4)	0.59*
	Haemorrhage	1	3(1–4)	3(1–4)	0.37*
	Oedema	1	2(1–4)	2(1–4)	0.24
	Necrosis	1(1–2)	3(1–4)	4(1–4)	0.44*
	Thrombosis	1	1(1–4)	2(1–4)	0.34*
Dermis—scale pockets	Absence of scale pockets	1(1–2)	1(1–3)	3(1–4)	0.65*
	Pocket inflammatory infiltrate	1(1–2)	3(2–4)	4(1–4)	0.47*
	Pockets with oedema/fibrin	1(1–4)	2(1–4)	2(1–4)	0.23
	Scales in re‐absorption phase	3(1–3)	3(1–4)	1(1–4)	−0.43*
	Inflammatory infiltrate below scales	1(1–2)	2(1–4)	1(1–4)	−0.27
	Absence of scales	1	3(1–4)	4(2–4)	0.84*
	Scale regeneration	1	1	1(1–4)	0.33*
	Multinucleated cells	1	1	1(1–4)	0.41*
Dermis—stratum compactum	Cellular infiltration	2(1–3)	3(2–4)	4(3–4)	0.66*
	Vessel congestion	1	2(1–4)	3(1–4)	0.45*
	Haemorrhage	1	2(1–4)	2(1–4)	0.31*
	Oedema	1	1(1–4)	1(1–3)	0.20
	Thickening	1(1–2)	3(1–4)	4(2–4)	0.58*
	Loss of structure	1(1–3)	3(1–4)	4(2–4)	0.67*
Hypodermis (subcutaneous tissue)	Cellular infiltration	3(1–3)	3(2–4)	4(2–4)	0.48*
	Vessel congestion	1	2(1–4)	2(1–4)	0.27
	Haemorrhage	1	2(1–4)	1(1–4)	0.10
	Enlarged lymphoid vessels	1	1(1–3)	1(1–4)	0.26
	Granulomatous reaction surrounding vessels and nervous fibres	1	2(1–3)	4(1–4)	0.67*
	Multinucleated cells	1	1	1(1–4)	0.37*
Muscle	Lymphocyte infiltration	1(1–3)	3(2–4)	4(2–4)	0.62*
	Granulomatous reaction surrounding the myofibres	1	2(1–4)	3(1–4)	0.56*
	Miosepta infiltration	1(1–3)	2(1–4)	3(1–4)	0.49*
Peritoneal serosa	Lymphocyte infiltration	1(1–2)	2(1–4)	2(1–2)	0.35
	Visceral adhesions	1	1(1–4)	1	0.04

Note

For each parameter, the score ranged from 1 to 4, as follows: 1 = absence of the lesion; 2 = mild lesion; 3 = moderate lesion; and 4 = severe lesion. Legend: median (min–max).

* *p* < .05.

### 2.4 Proliferating cell nuclear antigen (PCNA) immunohistochemistry

Twenty skin/muscle samples, representative of the scored lesions (6 macroscopically scored as type I, 5 as type II and 9 as type III), were evaluated with immunohistochemistry using an anti‐PCNA antibody to assess the cell proliferation rate within the lesion. Four‐micron‐thick formalin‐fixed, paraffin‐embedded sections were deparaffinized for 30 min in Solvent Plus (Carlo Erba) and then hydrated in a series of graded alcohols. Subsequently, endogenous peroxidase activity was blocked with 3% hydrogen peroxide in distilled water for 30 min at room temperature; sections were then treated in citrate buffer (pH 6.0) for 10 min in a microwave oven (750 W) for antigen retrieval. Non‐specific binding was blocked by incubating the sections with 5% normal goat serum and 1% bovine serum albumin (BSA) and phosphate‐buffered saline (PBS) (blocking solution) for 1 hr at room temperature, and then, sections were incubated overnight at 4°C with mouse monoclonal anti‐PCNA (Clone PC10, Cell Signaling Technology) diluted 1:4,000 in blocking solution. A horseradish peroxidase (HRP) polymer conjugate SuperPicture™ Detection Kit (Zymed, Invitrogen) was applied to the sections for 10 min at room temperature to visualize antibody–antigen binding. The immunohistochemical reaction was developed with 3,3‐diaminobenzidine solution (Sigma‐Aldrich). The slides were counterstained with Papanicolaou haematoxylin, dehydrated in a series of graded alcohols and cleared in xylene before mounting.

### 2.5 Semi‐quantitative evaluation of PCNA‐positive cells

For each stained section, the percentage of PCNA‐positive cells was evaluated (RS, LM) in the epidermis (epithelial cells), the dermis (lymphocytes, endothelial cells and fibroblasts) and the hypodermis (lymphocytes, endothelial cells and fibroblasts) considering four score levels: 0 (negative), 1 (positivity range: 1%–25%), 2 (positivity range: 25%–50%), 3 (positivity range: 50%–75%) and 4 (positivity range: 75%–100%).

### 2.6 Statistical analysis

Score data of skin histological variables, listed in Table 1, and score of PCNA positivity were tested for normality using the Shapiro–Wilk W test. Because the data distribution was not normal, the correlation of the scored variables with the three types of gross lesions was performed using the non‐parametric Spearman rank‐order correlation test (CSS Statistica, Statsoft). A *p* value <.05 was considered significant.

## 3 RESULTS

### 3.1 Gross skin lesions

**FIGURE 1:**
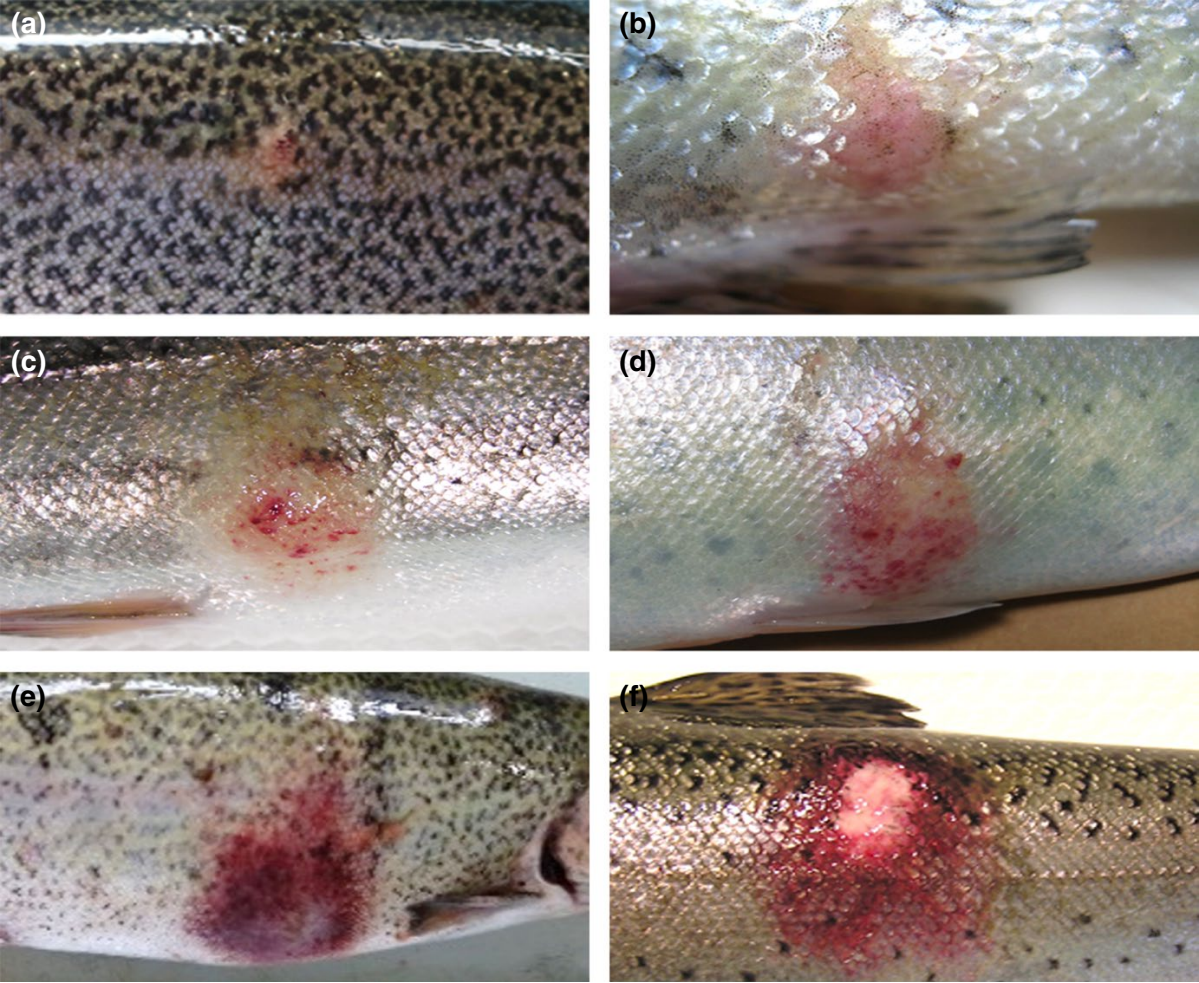
Classification of RMS skin lesions by macroscopic features into three categories. (a–b) Type I lesions, consisting of small up to 1 cm flat macules, with discoloration, mild exudate and visible scales; (c–d) type II lesions, characterized by slightly raised 1–2 cm patches, covered by clear exudate associated with multifocal petechiae and slight scale loss; (e–f) type III lesions,consisting of slightly raised large patches, more than 2 cm, covered by serous/fibrin exudate with marked redness and haemorrhages, evident scale loss and area of erosion

Lesions were allocated to one of three categories as follows: type I = 7 lesions (Figure 1a, b), type II = 19 lesions (Figure 1c, d) and type III = 20 lesions (Figure 1e, f). The macroscopic examination of visceral organs did not reveal lesions apart from a slightly increased spleen volume in 18 out of 46 (39%) sampled trout. These spleens also showed a rough surface, and upon dissection, they showed congestion.

### 3.2 Histology

The histological observation of skin sections allowed describing the main features of each gross lesion type, as follows:

#### 3.2.1 Type I

**FIGURE 2:**
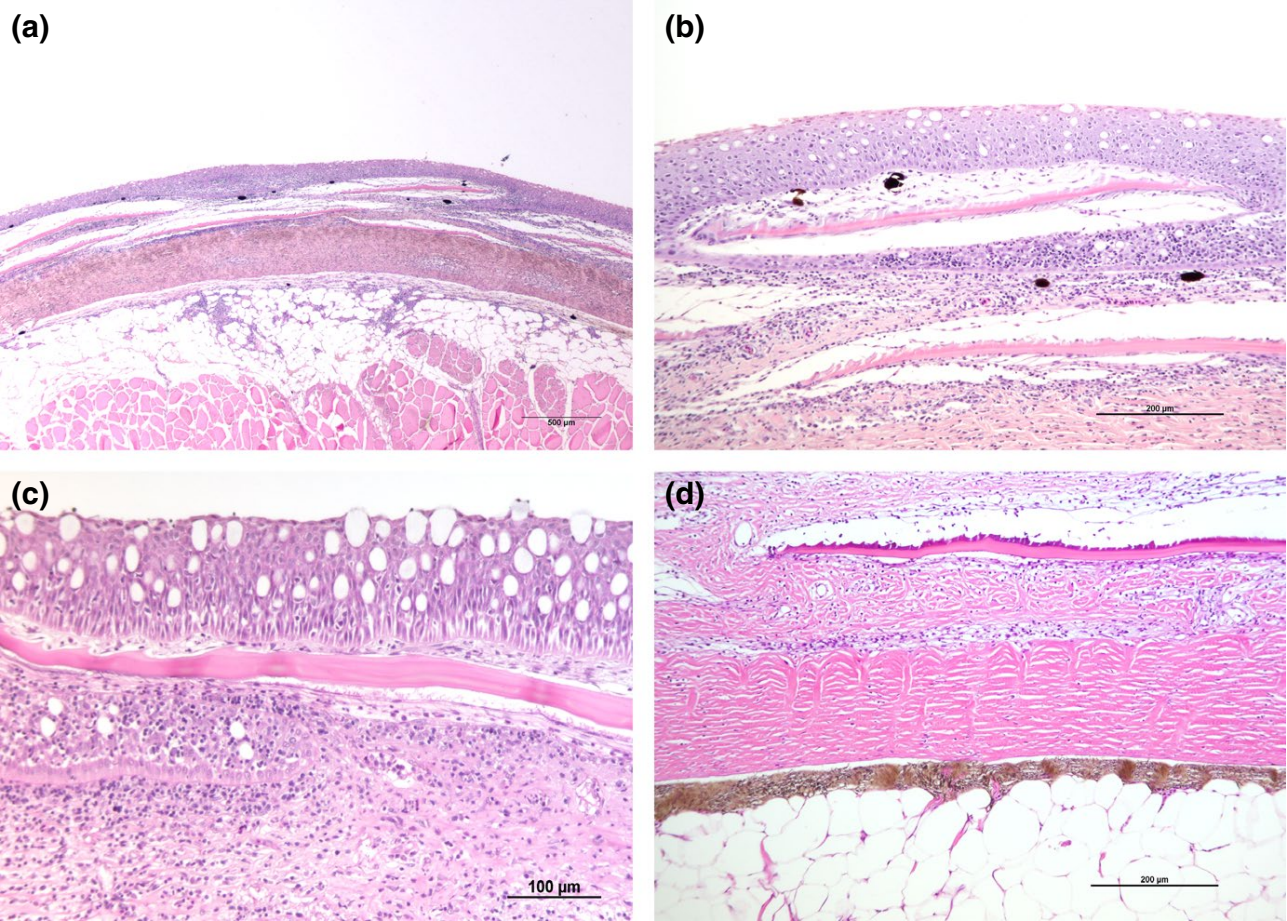
Histological features of the type I lesion. (a) Histology revealing a slight inflammation involving all the skin layers, including the hypodermis. The epidermis is intact. The scale pockets and scales are present, with a slight cellular infiltration (H&E). (b) Detail of Figure 2. The epidermis is still intact and the *stratum spongiosum* shows a moderate cellular infiltration, particularly evident under the scales (H&E). (c) Detail of the epidermis and *spongiosum* layer of the dermis. The epidermis is intact and unaffected by inflammatory infiltrate, otherwise a moderate mononuclear infiltrate is observed in the *spongiosum* layer, mainly around scale pockets. (H&E). (d) Detail of the *spongiosum* layer in dermis, affected by a cellular inflammatory infiltrate. The compact layer and the hypodermis are unaffected (H&E)

Mild gross skin lesions. The epidermis was always intact, only rarely showing areas with slight intra‐epithelial lymphocyte exocytosis. The single constant observation was a mild‐to‐moderate infiltration of lymphocytes and monocytes in the SS of the dermis, involving also the area surrounding the SP. The dermal SC in some samples (5 out of 7, 71%) appeared with a mild‐to‐moderate lymphocyte infiltration, whereas the hypodermis revealed a moderate infiltration of lymphocytes and monocytes (Figure 2).

#### 3.2.2 Type II

**FIGURE 3:**
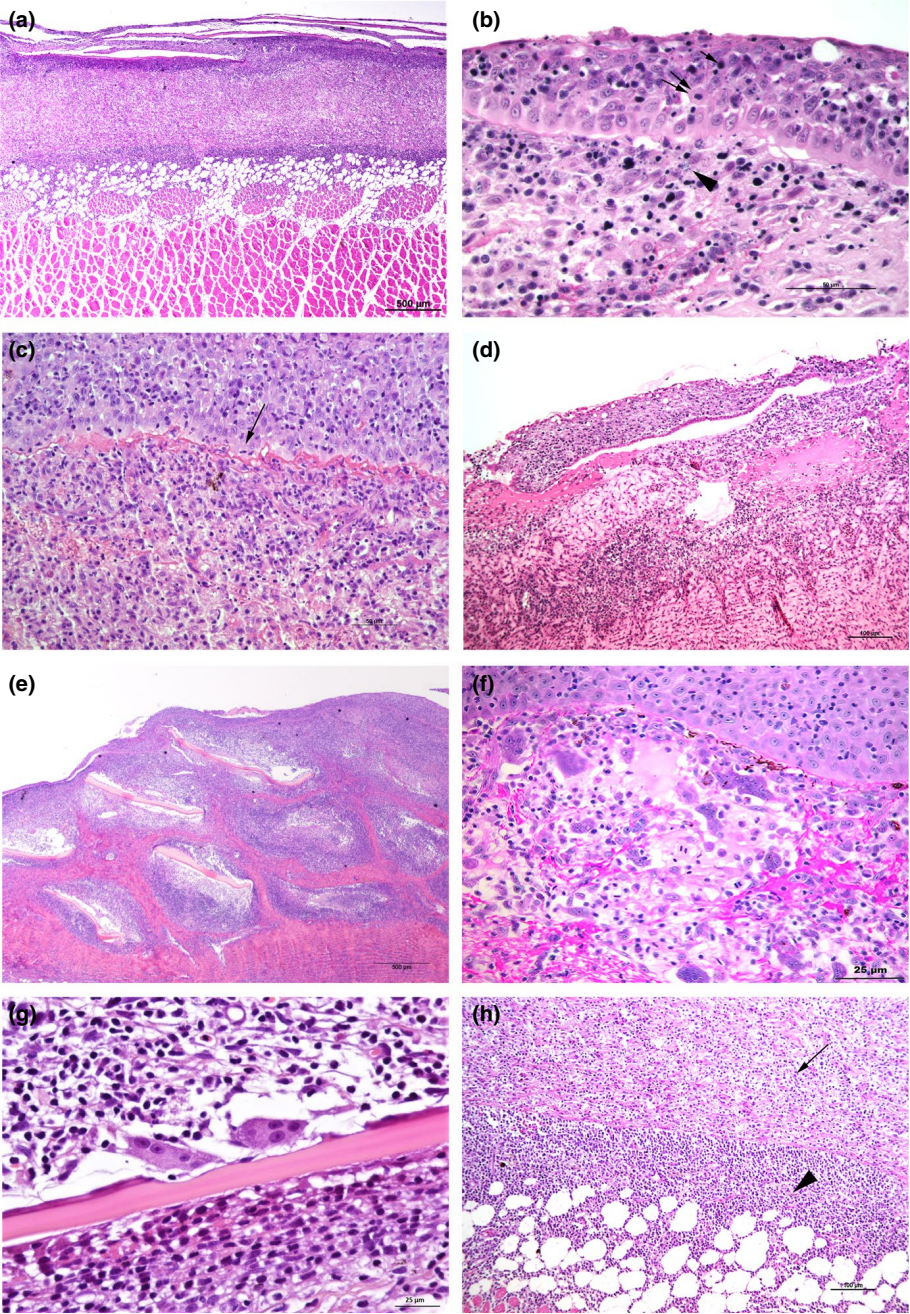
Histological features of the type II lesion. (a) Histology revealing an inflammatory response involving all the skin layers from epidermis to hypodermis, until the underlying muscular tissue. The *stratum spongiosum* appears moderately infiltrated, mainly in the areas below the scales, which are still intact. The *stratum compactum* of the derma appears thickened with a moderate inflammatory infiltrate, recruited also towards the hypodermis and the muscular layer (H&E). (b) Detail of epidermis, early spongiosis, nuclei pyknosis (double arrow) and some infiltrating lymphocytes (single arrow) among necrotic cells. Karyorrhectic cells (arrowhead) can be observed along a mild monocyte–macrophage infiltrate in the underlying *stratum spongiosum*, in the proximity of the dermo‐epidermal junction (H&E). (c) Marked epidermis thickening with a discrete inflammatory infiltrate and cell necrosis. The dermo‐epidermal junction shows extended thickening of the basement membrane (arrow). Moderate‐to‐severe inflammatory infiltrate and necrosis in the *derma spongiosum* (H&E). (d) Severe infiltrate by mononuclear cells at the dermo‐epidermal junction, with extensive necrosis characterized by abundant eosinophilic material. Evident lichenoid tissue reaction with epidermal detachment (H&E). (e) *Stratum spongiosum* shows scale pockets massively enlarged, with severe lymphocytes and monocyte infiltrate, and abundant acidophilic necrotic debris. Scales are either lacking or, when present, are under re‐absorption (H&E). (f) Scale pocket with numerous multinucleated osteoclasts. The infiltrate includes macrophages and presence of acidophilic fibrillar (necrotic) or pale (oedema) material (H&E). (g) Close‐up of a scale within its pocket showing an area of re‐absorption determined by osteoclasts. Note abundant infiltrate by mononuclear cells both above and below the scale (H&E). (h) Transition area between the *stratum compactum* (arrow) and the hypoderma (arrowhead). Dermatitis and panniculitis with widespread and severe infiltration of lymphocytes, plasma cells and monocytes, both in the *stratum compactum* and in the hypodermis, determining a loss of architecture (H&E)

Moderate gross skin lesions. There was moderate inflammation involving all the skin layers from the epidermis to the underlying muscular tissue (Figure 3a). The epidermis was often present or partially missing. In some specimens (7 out of 19, 37%), a multifocal epidermal necrosis or epidermal oedema with acanthosis was evident (Figure 3b). Multifocal mild lymphocyte intra‐epithelial infiltration was observed (Figure 3b). A lichenoid inflammatory pattern was observed, characterized by monocyte and lymphocyte infiltration in the dermo‐epidermal junction, sometimes associated with epidermal layer detachment (Figure 3d). The dermo‐epidermal junction showed in some cases extended thickening of the basement membrane (Figure 3c). The SS was affected by moderate‐to‐severe infiltration of lymphocytes and monocytes as well as occasional recruitment of neutrophils and constant oedema coupled with lymphatic dilatation. SPs were frequently affected by a moderate‐to‐severe infiltration of lymphocytes, monocytes and macrophages distributed internally throughout the pockets or below the scales (Figure 3e). Sometimes the SPs were filled with pale eosinophilic fluid, which caused a marked oedema. Occasionally, the scales were partially or fully degenerated with multinucleated osteoclasts involved in scale resorption (Figure 3f, g). The SC appeared thickened by about a third compared with the normal thickness, with moderate‐to‐severe infiltration of lymphocytes (Figure 3h), slight haemorrhages and oedema. The hypodermis showed panniculitis with a moderate‐to‐severe infiltration of lymphocytes, plasma cells and macrophages (Figure 3h). A mild fibrosis was also detectable. The underlying muscular tissue showed moderate‐to‐severe infiltration of lymphocytes and plasma cells, with frequent mitoses. Muscle inflammation was mainly localized beneath the areas of panniculitis and mainly involved the myosepta. In a few cases, both hypodermal tissue inflammation and muscular tissue inflammation showed a granulomatous pattern. Inflammation was sometimes observed in deeper areas, along the intramuscular septa and up to the peritoneum.

#### 3.2.3 Type III

**FIGURE 4:**
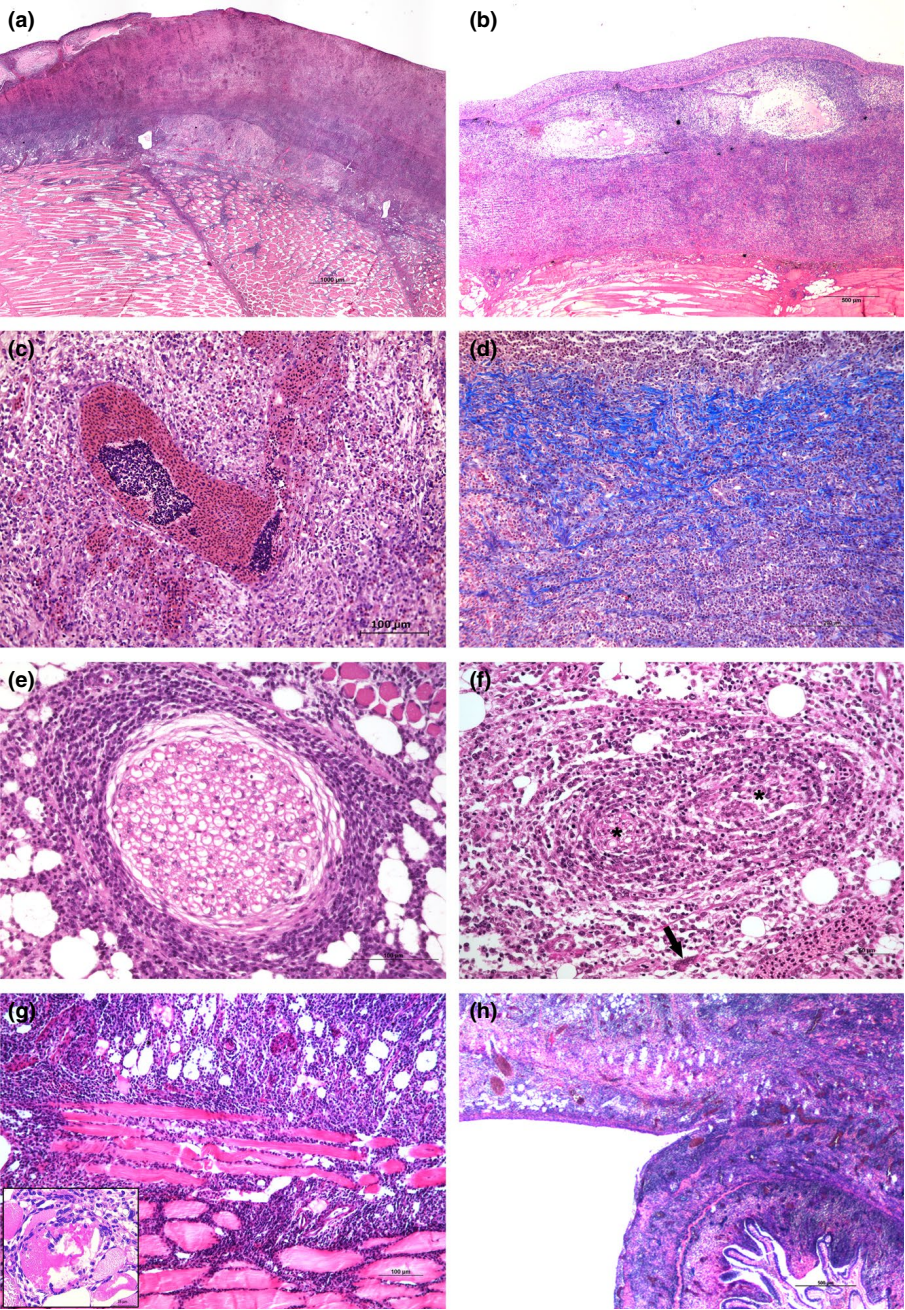
Histological features of the type III lesion. (a) Severe cellular infiltration involving all the layers from epidermis down to the muscular tissue with epidermis partially missing. The *stratum spongiosum* is thinned, and no scales can be observed. The dermal *stratum compactum* appears thickened and severely infiltrated, deeply modifying the layer's architecture. Severe infiltration reaches the hypodermis (panniculitis) and the underlying muscular layers (myositis), spreading also between the myosepta (H&E). (b) Severe cellular infiltration pattern involving all skin layers. The epidermis is still present but subjected to lymphocyte exocytosis. Scales are absent in the two scale pockets of the *stratum spongiosum* replaced by severe oedema and influx of inflammatory cells. The *stratum compactum* is markedly thickened and heavily infiltrated (H&E). (c) High magnification of a strongly congested venous vessel in the *stratum spongiosum*, with the development of luminal thrombi and surrounded by severe infiltration (H&E). (d) Evident thickening of the skin layer due to severe and widespread cellular infiltration (monocytes, macrophages, lymphocytes and plasma cells) of the connective fibres matrix of the *stratum compactum* that modifies the tissue structure (Van Gieson Weigert trichrome). (e) Severe infiltration of lymphocytes and plasma cells surrounding nerve fibres observed as a particular feature of the panniculitis. The reaction includes a fibro‐connective tissue reaction around the perineurium (H&E). (f) Granulomatous reaction surrounding residual nerve fibres (asterisks) includes numerous monocytes–macrophages, lymphocytes, histiocyte‐like cells and, occasionally, multinucleated giant cells of the syncytial type (arrow) (H&E). (g) Presence of a severe lymphocyte–plasma cell infiltrate between the myofibres, inducing atrophy and loss of architecture. Insert: details of a degenerated myofibre with internal macrophages undergoing phagocytosis (H&E). (h) Fibrous adhesion reaction between the serosa and the gut wall was observed in section of the abdominal wall, intestine adherent to the serosa/muscular layer. Note inflammation in this case reaches the peritoneal serosa (H&E)

Severe gross skin lesions. A severe inflammation involving all the skin layers from the epidermis down to the underlying muscular tissue was always present (Figure 4a). The epidermis was absent in 10 out of 20 (50%) skin sections due to erosion and ulceration of the epithelium (Figure 4a). When the epidermis was present, necrosis and severe intra‐epithelial lymphocyte infiltration were observed. At the dermo‐epidermal junction, cells necrosis, capillary congestion and haemorrhages were observed. In the case of skin ulceration, the lesion involved the epidermis, the SS and partially the SC. The SS contained numerous necrotic cells and a severe inflammatory infiltrate composed of lymphocytes, macrophages and scarce neutrophils. Vascular congestion was always severe, accompanied by moderate haemorrhages and slight oedema. In some samples, the vessels displayed thrombosis (Figure 4c). Scales were absent due to complete resorption, and in some specimens, an early process of regeneration was detected. The SPs were numerically reduced or lacking, replaced by areas of chronic inflammation (recruitment of lymphocytes, plasma cells, macrophages and multinucleated giant cells) (Figure 4b). The SC displayed a severe cellular infiltrate including monocytes, macrophages, lymphocytes and plasma cells. Inflammatory cells spread between the connective fibres, modifying the tissue structure and inducing marked thickening of the skin layer as shown by Van Gieson Weigert trichrome staining (Figure 4d). A strong vascular congestion was often seen. Panniculitis often presented a granulomatous pattern, mainly concentrated around small vessels and nerve fibres (Figure 4e, f). In samples where the granulomatous pattern was more evident, multinucleated giant cells were also observed (Figure 4f). A similar pattern was present in the underlying muscular tissue. Necrosis of the myofibrils was often observed and associated with macrophages engulfing necrotic muscular tissue debris (Figure 4g). Within this histological pattern, several infiltrating cells were apoptotic. Occasionally, the phlogosis reached the peritoneal serosa, mainly in specimens where the gross skin lesions affected the flank or the ventral area. In these specimens, fibrous adhesions were detected between the serosa and the gut wall (Figure 4h).

Lesions affecting the internal organs were observed in trout with type II and type III lesions. Among the internal organs, histological lesions were observed in the heart, spleen, kidney and liver. In 19 out of 46 examined fish, heart lesions included pericarditis (17 fish, 37%) and myocarditis (2 fish, 4%), both with lymphocyte infiltration. In the spleen, hyperplasia of the white pulp associated with perivascular histiocytic cell proliferation was observed in 18 out of 46 fish (39%). The liver showed foci of lymphocyte infiltration and lympho‐histiocytic vasculitis in 13 out of 46 specimens examined (28%). In 14 out of 46 kidney specimens (30%), a mild vacuolar degeneration of tubular epithelium was observed. No pathological changes were observed in the gill, brain and gut samples.

### 3.3 Data analysis by statistics

The correlation between the single histological scored parameter and the three types of gross lesions, assessed with the Spearman rank‐order correlation, revealed several significant results but with different conclusive considerations (Table 2). A significant positive correlation (score increase) from type I to type III gross lesions was apparent with the following histological parameters (supplementary graphs a–f): progressive loss of the epithelium and scales, progressive recruitment of inflammatory cells in the SC of the dermis, loss of regular architecture of the SC of the dermis and increase in granuloma‐like reactions surrounding the small vessels and muscular fibres (highest *R* value in Table 2). The above‐mentioned variables thus acquired a higher score parallel to the progression of the macroscopic lesion. For several other variables, the results showed lower values in the type I group compared with the other groups, in which type II was similar to type III. These variables did not provide any indication of the progression of the lesion from type I to type III gross features; if scored with lower values, they only offered an indication of an early and mild gross feature.

### 3.4 Immunohistochemistry

**FIGURE 5:**
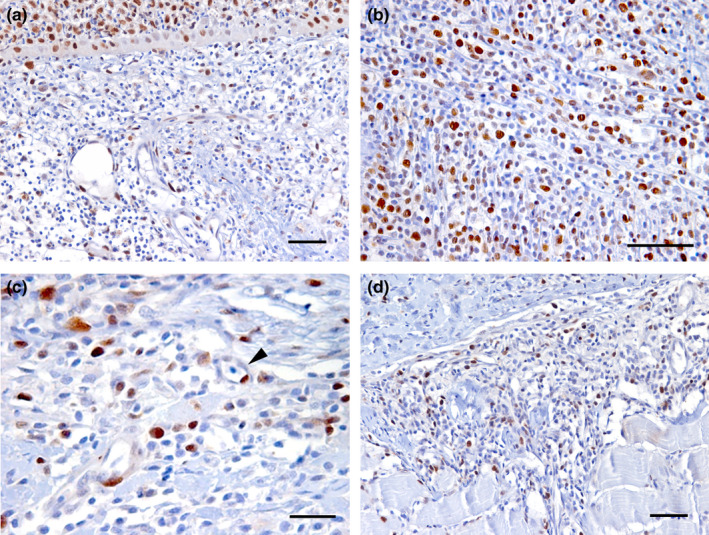
Immunohistochemical stain to PCNA. (a) Positive nuclear stain to PCNA in epidermis (top of the figure) and dermis, in this latter localized mainly in lymphocytes. (b) Higher magnification of dermal‐positive nuclear stain to PCNA. (c). Positive nuclear stain to PCNA in vessels (arrowhead). (d) Subcutis showing positive PCNA lymphocytes. Bar: a, b, d = 50 micron; c = 25 micron. All images from type 3 lesions

Several PCNA‐positive cells were found in the epidermis (lymphocytes) (Figure 5a, b) and in the dermis (lymphocytes, fibroblasts and endothelial cells) (Figure 5c), but only PCNA‐positive lymphocytes (Figure 5d) were found in the hypodermis. A significant increase in PCNA‐positive lymphocytes from gross type I to gross type III cases was revealed only in the hypodermis (supplementary graph g); no other association was apparent.

## 4 DISCUSSION

The clinical evaluation and the differential characterization of the skin lesions examined in the present study were based on the RMS case definition in rainbow trout proposed by Oidtmann et al. (2013). The cases analysed here were compatible with RMS considering the criteria suitable for the definition of RMS outbreaks such as affected fish size, water temperature, clinical signs, gross lesion appearance and distribution and histopathological features. Moreover, part of the skin lesion samples analysed confirmed the presence of intracytoplasmic microorganisms resembling Rickettsiales within macrophages, fibroblasts and erythrocytes by TEM, as described previously by Galeotti, Manzano, et al. 2017. From a macroscopic point of view, the skin lesions of the cases described here do not differ from those observed in the United States as USSD (Lloyd et al., 2008; Olsen et al., 1985) and in other European countries (Ferguson et al., 2006;, Oidtmann et al., 2006, 2013; Noguera et al., 2008; Rodger, 2008; Schmidt et al., 2017; Schimidt‐Posthaus et al., 2009) or Turkey (Kubilay et al., 2014).

From the results of histological evaluations, type I lesions are characterized by an always intact epidermal layer, a slight lymphocyte and monocyte infiltration in the dermal SS, also involving the area surrounding the SP, and a scarce presence or complete absence of neutrophils. Type II lesions are characterized by inflammation involving all the skin layers, from the epidermis to the underlying muscular tissue, a moderate‐to‐severe inflammatory reaction on the scales and the SP with mononuclear cell infiltration, both within the pockets and right below the scales. The scales are partially or fully degenerated with multinucleated osteoclasts, which are involved in scale resorption. The hypodermis shows a moderate infiltration of mononuclear cells. In type III lesions, scales are absent due to complete resorption, the SC is markedly thickened with loss of normal structure due to evident lymphocyte and monocyte infiltration and myofibril necrosis is often observed. These findings are consistent with those reported previously by other authors (Ferguson et al., 2006; Fleury et al., 1985; Kfoury et al., 1996; Kubilay et al., 2014; La Patra et al., 1994; Lloyd et al., 2008; McCarthy et al., 2013; Metselaar et al., 2010; Noguera et al., 2008; Olson et al., 1985; Rodger, 2008; Sasani et al., 2016; Sandoval et al., 2016; Schmidt Posthaus et al., 2009; Verner‐Jeffreys et al., 2008).

The term “panniculitis” denotes the localization of inflammation to the fat of the subcutis. From a comparative point of view, the existence of a similar stratigraphy (skin/subcutis/muscle) in mammals and fish allows the use of the term “panniculitis” in fish. It results from primary damage of subcutis or by extension from surrounding layers, mainly secondary to dermatitides. Panniculitis from any cause is enhanced by lipid release from damaged lipocytes because lipids are vigorous stimulators of inflammation (Gross et al., 2005).

Adding to the current state of knowledge, the results of the present work contribute particularly with the observations of type II and type III lesions; these data highlight that panniculitis and myositis may display a granulomatous pattern surrounding vessels as well as the nerves and muscle fibres. This pattern, when observed in the hypodermis, can be defined as panniculitis referring to the development of severe granulomatous reactions in the proximity of blood vessels and nerve fibres. This condition is consistently associated with severe myositis of the underlying tissue. Furthermore, in some samples, the chronic inflammatory reaction also reaches the peritoneal serosa, with adhesions developing between the serosa and the gut wall. Furthermore, the SS of type III lesion in some samples shows vessels with occlusive thrombosis and early processes of scale regeneration, thus demonstrating that this regeneration occurs not only in fully healing injuries but also in ongoing injuries.

At the internal organ level, the most relevant microscopic lesions were observed in the heart and spleen. The pericarditis observed in 17 out of 46 trout affected by RMS represented the most prominent lesion, characterized by an intense lymphocytic infiltration of the pericardium. Similar findings were described by Ferguson et al., (2006), who reported heart lesions in 40% of *SD*‐affected fish (20% of which showed diffuse myocarditis) and by Verner‐Jeffreys et al., (2008), who reported epicarditis and cardiomyositis in 20% of the examined fish with RMS. In the spleen, hyperplasia of the white pulp associated with perivascular histiocytic cell proliferation was observed in 18 out of 46 individuals (39%), as also described by Metselaar et al., (2020). In 13 out of 46 trout examined, the liver showed foci of lymphocyte infiltration and vasculitis. The observation of liver focal necrosis was also recorded in RMS‐affected fish by Noguera et al., (2008) and Verner‐Jeffreys et al., (2006). Mild tubular degeneration in the kidney was observed in 14 out of 46 specimens, similarly to findings by Noguera et al., (2008) and Verner‐Jeffreys et al., (2006), whereas a moderate‐to‐severe diffuse histiocytic proliferation of the interstitial haematopoietic tissue was also reported by Metselaar et al. (2010) and Metselaar et al. (2020).In the gill, brain and gut, no pathological changes were observed. All the lesions in the internal organs were recorded in trout showing macroscopic lesions classified as type II or type III, suggesting a systemic compromise.

The observations made in this study and the statistical data analysis allowed us to survey the evolution of the histological lesion and to define three macroscopic stages identifying the most relevant phases of the disease progression. The correlation between single histologically scored parameters with the three gross lesion types revealed meaningful correlations for some variables. A significant positive correlation (score increase) from type I to type III gross lesions was apparent in the case of six parameters: progressive loss of the epithelium, progressive loss of the SP, progressive recruitment of inflammatory cells in the SC of the dermis, loss of regular architecture of the SC of the dermis, increase in granuloma‐like reactions surrounding the small vessels and nerve fibres of the hypodermis and in the muscular fibres. The above‐mentioned variables thus acquire a higher score, parallel to the progression of the macroscopic lesion. The observation of these lesions together with the recovery of vessels displaying occlusive thrombosis in SS indicates a clear aggravation of the process in the skin. It is also appropriate to take into consideration the observation of pericarditis and of splenic reactivity. These data could be used for an objective assessment of the aggravation and systemic spread of the process, beyond the morphological aspect of the external lesions.

Regarding some possible insights on the disease pathogenesis, our observations are in line with those reported by Ferguson et al., (2006). Skin lesions display aspects similar to the lichenoid dermatitis observed and described in humans and other mammals as a result of the T‐cell‐mediated or autoimmune response (Deschaine & Lehman, 2019; Gross et al., 2005). The strong presence of lymphocytes and macrophages, also reported by McCarthy et al., (2013) and von Gersdorff Jørgensen et al., (2019), suggests an immunopathological mechanism promoting the development of these lesions. In our study, the presence of microthrombosis and necrosis of the epidermis observed in advanced severe lesions is similar to that recorded in skin inflammatory reactions caused by the accumulation of immune complexes (type III), which can coexist with forms of delayed‐type hypersensitivity (type IV), as happens in canine scabies, where type III and type IV mechanisms coexist (Mauldin & Peters‐Kennedy, 2016). Furthermore, the fact that skin lesions caused by RMS easily tend to recover spontaneously reinforces the hypothesis of an immune‐mediated disease, as observed in serum sickness, an experimental animal model of human immunological disease (Lawley et al., 1984; Rixe et al., 2020). Recent studies on RMS pathogenesis have suggested the involvement of a delayed‐type Th‐1 cell‐mediated immune response with a concurrent high production of immunoglobulins IgD, IgM and IgT, possibly independent from the Th‐2 pathway (von Gersdorff Jørgensen et al., 2019).

Concerning the immunohistochemical detection of PCNA positivity, it is interesting to note how positive proliferating cells were found in the epidermis (only lymphocytes), the dermis (lymphocytes, fibroblasts and endothelial cells) and the hypodermis (only lymphocytes). A significant increase in PCNA‐positive lymphocytes from type I to type III lesions was only observed in the hypodermis. The proliferation model in endothelial cells (which indicates angiogenesis) and in fibroblasts (which indicates tissue repair) is consistent with an inflammatory reaction localized in the dermis, whereas the presence of proliferating lymphocytes, associated with granulomatous inflammation, indicates lymphocyte activation in the hypodermis.

In conclusion, in the light of our experience with the histological observation of skin lesions from 46 RMS‐affected trout, classified in three gross developmental stages, and based on the statistical analysis of the histological scores, we suggest an improvement of the initial first accurate descriptions of the “Strawberry disease” skin lesion “…focal, non‐suppurative dermatitis with ulceration and extensive infiltration of mononuclear inflammatory cells” (Olson et al., 1985), proposing a new morphological diagnosis as “…deep chronic dermatitis associated to panniculitis and myositis, characterised by lympho‐histiocytic and granulomatous reaction.”

## CONFLICT OF INTEREST

The authors declare that there are no conflicts of interest.

## Supporting information

Supplementary Material

## Data Availability

The data supporting the findings of this study are available from the corresponding author upon request.

## References

[jfd13391-bib-0001] Bruno, D., Crumlish, M., LaPatra, S., Noguera, P., & Verner‐Jeffreys, D. (2007). Workshop on salmonid skin diseases. In: European Association of Fish Pathologists 13th International Conference on Fish and Shellfish Diseases.

[jfd13391-bib-0002] Cafiso, A., Sassera, D., Serra, V., Bandi, C., McCarthy, U., & Bazzocchi, C. (2015). Molecular evidence for a bacterium of the family Midichloriaceae (order Rickettsiales) in skin and organs of the rainbow trout *Oncorhynchus mykiss* (Walbaum) affected by red mark syndrome. Journal of Fish Diseases, 39, 497–501. 25828398 10.1111/jfd.12371

[jfd13391-bib-0003] Deschaine, M. A., & Lehman, J. S. (2019). The interface reaction pattern in the skin: An integrated review of clinical and pathological features. Human Pathology, 91, 86–113. 10.1016/j.humpath.2019.06.004 31278974

[jfd13391-bib-0004] Ferguson, H. W., Girons, A., Rizgalla, G., LaPatra, S., Branson, E. J., Mackenzie, K., Davies, M., Collins, R. O., Diab, A., & Crumlish, M. (2006). Strawberry disease in rainbow trout in Scotland: Pathology and association with Flavobacterium psychrophilum. Veterinary Record, 158, 630–632. 10.1136/vr.158.18.630 16679482

[jfd13391-bib-0005] Fleury, H. J. A., Vuillaume, A., & Sochon, E. (1985). Isolation of an adeno‐like virus from two cases of strawberry disease in rainbow trout. Annales De L'institut Pasteur Virology, 136, 223–228. 10.1016/S0769-2617(85)80072-1

[jfd13391-bib-0006] Galeotti, M., Giavenni, R., Volpatti, D., Beraldo, P., & Feist, S. W. (2011). Red mark syndrome/cold water strawberry disease: Emergence in Italy and histopathological investigations. In: 15th International Conference of the European Association of Fish Pathologists.

[jfd13391-bib-0007] Galeotti, M., Manzano, M., Beraldo, P., Bulfon, C., Rossi, G., Volpatti, D., & Magi, G. E. (2017). Ultrastructural and biomolecular detection of Rickettsiales‐like organisms in tissues of rainbow trout with Red Mark Syndrome. Journal of Fish Disease, 40(7), 907–917. 10.1111/jfd.12571 27882570

[jfd13391-bib-0008] Galeotti, M., Ronza, P., Beraldo, P., Bulfon, C., Magi, G. E., Manzano, M., & Volpatti, D. (2017). First report of Red Mark Syndrome (RMS) in farmed rainbow trout in Slovenia. Journal of Fish Diseases, 40(12), 1935–1939. 28548687 10.1111/jfd.12652

[jfd13391-bib-0009] Galeotti, M., Volpatti, D., Beraldo, P., Brunetti, B., Galletti, E., & Feist, S. W. (2013). Red Mark Syndrome in rainbow trout (*O. mykiss*) farmed in Italy: Anatomohistopathological investigations. Journal of Comparative Pathology, 148(1), 54.

[jfd13391-bib-0010] Galeotti, M., Volpatti, D., Byadgi, O., Beraldo, P., Orioles, M., Sarti, M., & Magi, G. E. (2021). Red Mark Syndrome (RMS) in farmed rainbow trout: First report of outbreaks in Bosnia and Herzegovina. Journal of Fish Disease, 44(5), 627–631. 10.1111/jfd.13336 33476400

[jfd13391-bib-0011] Gross, T. L., Ihrke, P. J., Walder, E. J., & Affolter, V. K. (2005). Skin diseases of the dog and cat (2nd ed.). Blackwell Science.

[jfd13391-bib-0037] Henriksen, N. H., & Schmidt, J. G.(2017). Red mark syndrome – A novel but serious problem in Danish rainbow trout fish farms. 18th International Conference of the European Association of Fish Pathologists, Belfast, UK.

[jfd13391-bib-0012] Kfoury, J. R., Okamoto, N., Tanaka, M., Yoshimizu, M., LaPatra, S. E., & Maita, M. (1996). “Rush” skin disease of rainbow trout. Fish Pathology, 31(49), 197–201, 12.

[jfd13391-bib-0013] Kubilay, A., Ciftci, S., Yildirim, P., Didinen, I., Metin, S., Demirkan, T., Ozen, M. R., & Oidtmann, B. (2014). First observation of Red Mark Syndrome (RMS) in cultured rainbow trout (*Oncorynchus mykiss* Walbaum, 1972) in Turkey. Bulletin of the European Association of Fish Pathologists, 34(3), 95–101.

[jfd13391-bib-0014] LaPatra, S. E., Groff, J. M., Lauda, K. A., Munn, B., & Jones, G. R. (1994). Dermatitides in cultured rainbow trout. In: International Symposium on Aquatic Animal Health, September 4‐8, 1994.

[jfd13391-bib-0015] Lawley, T. J., Bielory, L., Gascon, P., Yancey, K. B., Young, N. S., & Frank, M. M. (1984). A prospective clinical and immunologic analysis of patients with serum sickness. The New England Journal of Medicine, 311(22):1407–1413. 6387492 10.1056/NEJM198411293112204

[jfd13391-bib-0016] Lloyd, S. J., Lapatra, S. E., Snekvik, K. R., St‐Hilaire, S., Cain, K. D., & Call, D. R. (2008). Strawberry disease lesions in rainbow trout from southern Idaho are associated with DNA from a Rickettsia‐like organism. Diseases of Aquatic Organisms, 82, 111–118. 10.3354/dao01969 19149374

[jfd13391-bib-0017] Mauldin, E., & Peters‐Kennedy, J. (2016). Integumentary system. In: M. G. Maxie (Ed.). Jubb, Kennedy, and Palmer’s Pathology of Domestic Animals (6th ed., Vol. 1, pp. 673‐675). Elsevier.

[jfd13391-bib-0018] McCarthy, U., Casadei, E., Wang, T., & Secombes, C. J. (2013). Red mark syndrome in rainbow trout *Oncorhynchus mykiss*: Investigation of immune responses in lesions using histology, immunohistochemistry and analysis of immune gene expression. Fish & Shellfish Immunology, 34(5), 1119–1130. 10.1016/j.fsi.2013.01.019 23403161

[jfd13391-bib-0019] Metselaar, M., Thompson, K. D., Gratacap, R. M. L., Kik, M. J. L., Lapatra, S. E., Lloyd, S. J., Call, D. R., Smith, P. D., & Adams, A. (2010). Association of red‐mark syndrome with a Rickettsia‐like organism and its connection with strawberry disease in the USA. Journal of Fish Diseases, 33, 849–858. 10.1111/j.1365-2761.2010.01187.x 20854353

[jfd13391-bib-0020] Metselaar, M., Thompson, K. D., Paley, R., Green, D. M., Verner‐Jeffreys, D., Feist, S., & Adams, A. (2020). Investigating the involvement of a Midichloria‐like organism (MLO) in red mark syndrome in rainbow trout *Oncorhynchus mykiss* . Aquaculture, 528, 735485. 10.1016/j.aquaculture.2020.735485

[jfd13391-bib-0021] Noguera, P. (2008). Red Mark Syndrome (RMS). Fish Farmer, 31, 38.

[jfd13391-bib-0022] Oh, W. T., Giri, S. S., Yun, S., Kim, H. J., Kim, S. G., Kim, S. W., Kang, J. W., Han, S. J., Kwon, J., Jun, J. W., & Park, S. C. (2019). Emergence of Rickettsial Infection in Rainbow Trout (*Oncorhynchus mykiss*) Fry Displaying the Appearance of Red Mark Syndrome in Korea. Microorganisms, 7(9), 302. 10.3390/microorganisms7090302 PMC6780055 31470673

[jfd13391-bib-0023] Oidtmann, B., LaPatra, S. E., Verner‐Jeffreys, D., Pond, M., Peeler, E. J., Noguera, P. A., Galeotti, M., & Feist, S. W. (2013). Differential characterization of emerging skin diseases of rainbow trout ‐ a standardized approach to capturing disease characteristics and development of case definitions. Journal of Fish Diseases, 36(11), 921–937. 10.1111/jfd.12086 23448696

[jfd13391-bib-0024] Oidtmann, B., & Noguera, P. (2008). Red Mark Syndrome/Cold water strawberry disease. Epidemiological study. Finfish News, 6, 10–11.

[jfd13391-bib-0025] Olson, D. P., Beleau, M. H., Busch, R. A., Roberts, S., & Krieger, R. I. (1985). Strawberry disease in rainbow trout, Salmo gairdneri Richardson. Journal of Fish Diseases, 8, 103–111. 10.1111/j.1365-2761.1985.tb01191.x

[jfd13391-bib-0026] Rixe, N., Tavarez, M. M., & Serum Sickness . (2020). [Updated 2020 Apr 28]. In: StatPearls [Internet]. Treasure Island (FL): StatPearls Publishing; 2020 Jan. (Available from: https://www.ncbi.nlm.nih.gov/books/NBK538312/ 30855896

[jfd13391-bib-0027] Rodger, H. (2008). Red Mark Syndrome (RMS). Finfish News, 6, 8–10.

[jfd13391-bib-0036] Sandoval, C., Infante, J., Abad, J., Ferguson, H. W., Paredes, E., Valdebenito, S., Yáñez, A. J., Ilardi, P., & Avendaño‐Herrera, R. (2016). Case Report: Strawberry Disease in Farmed Chilean Rainbow Trout. J Aquatic Animals Health, 28(1), 1–10. 10.1080/08997659.2015.1114534 26913369

[jfd13391-bib-0028] Sasani, F., Shokrpoor, S., Rahmati‐Holasoo, H., & Zargar, A. (2016). Appearance of Red Mark Syndrome (RMS) in cultured rainbow trout (*Oncorynchus mykiss* Walbaum, 1972) in Iran. Bulletin of European Association of Fish Pathologists, 36(2), 90–94.

[jfd13391-bib-0029] Schmidt, J. G., Jørgensen, L., Chen, D., Buchmann, K., Iburg, T., & Olesen, N. (2017). Rainbow trout red mark syndrome lesion development visualized. In: 18th International Conference of the European Association of Fish Pathologists.

[jfd13391-bib-0030] Schmidt‐Posthaus, H., Bergmann, W., Knüsel, R., Heistinger, H., & Licek, E. (2009). Appearance of red mark syndrome/cold water strawberry disease in Switzerland and Austria. Diseases of Aquatic Organisms, 88, 65–68. 10.3354/dao02152 20183966

[jfd13391-bib-0032] St‐Hilaire, S., & Jeffery, K. (2004). Strawberry disease in rainbow trout. Trout News, 37, 24.

[jfd13391-bib-0033] Verner‐Jeffreys, D., Algoet, M., Feist, S., Bateman, K., Peeler, E., & Branson, E. (2006). Studies on Red Mark Syndrome. Finfish News, 1, 19–22.

[jfd13391-bib-0034] Verner‐Jeffreys, D. W., Pond, M. J., Peeler, E. J., Rimmer, G. S. E., Oidtmann, B., Way, K., Mewett, J., Jeffrey, K., Bateman, K., Reese, R. A., & Feist, S. W. (2008). Emergence of cold water strawberry disease of rainbow trout *Oncorynchus mykiss* in England and Wales: Outbreak investigations and transmission studies. Diseases of Aquatic Organisms, 79, 207–218. 10.3354/dao01916 18589997

[jfd13391-bib-0035] von Gersdorff Jørgensen, L., Schmidt, J. G., Chen, D., Kania, P. W., Buchmann, K., & Olesen, N. J. (2019). Skin immune response of rainbow trout (*Oncorhynchus mykiss*) experimentally exposed to the disease Red Mark Syndrome. Veterinary Immunology & Immunopathology, 211, 25–34. 10.1016/j.vetimm.2019.03.008 31084890

