# Supplementary figures and images for: Red mark syndrome of trout (Oncorhynchus mykiss; Walbaum, 1792): Histopathological scoring and correlation with gross lesions
Source: J Fish Dis. 2021 May 10;44(9):1325–36. doi: 10.1111/jfd.13391 (PMC8453541; doi:10.1111/jfd.13391)

## Slide 1
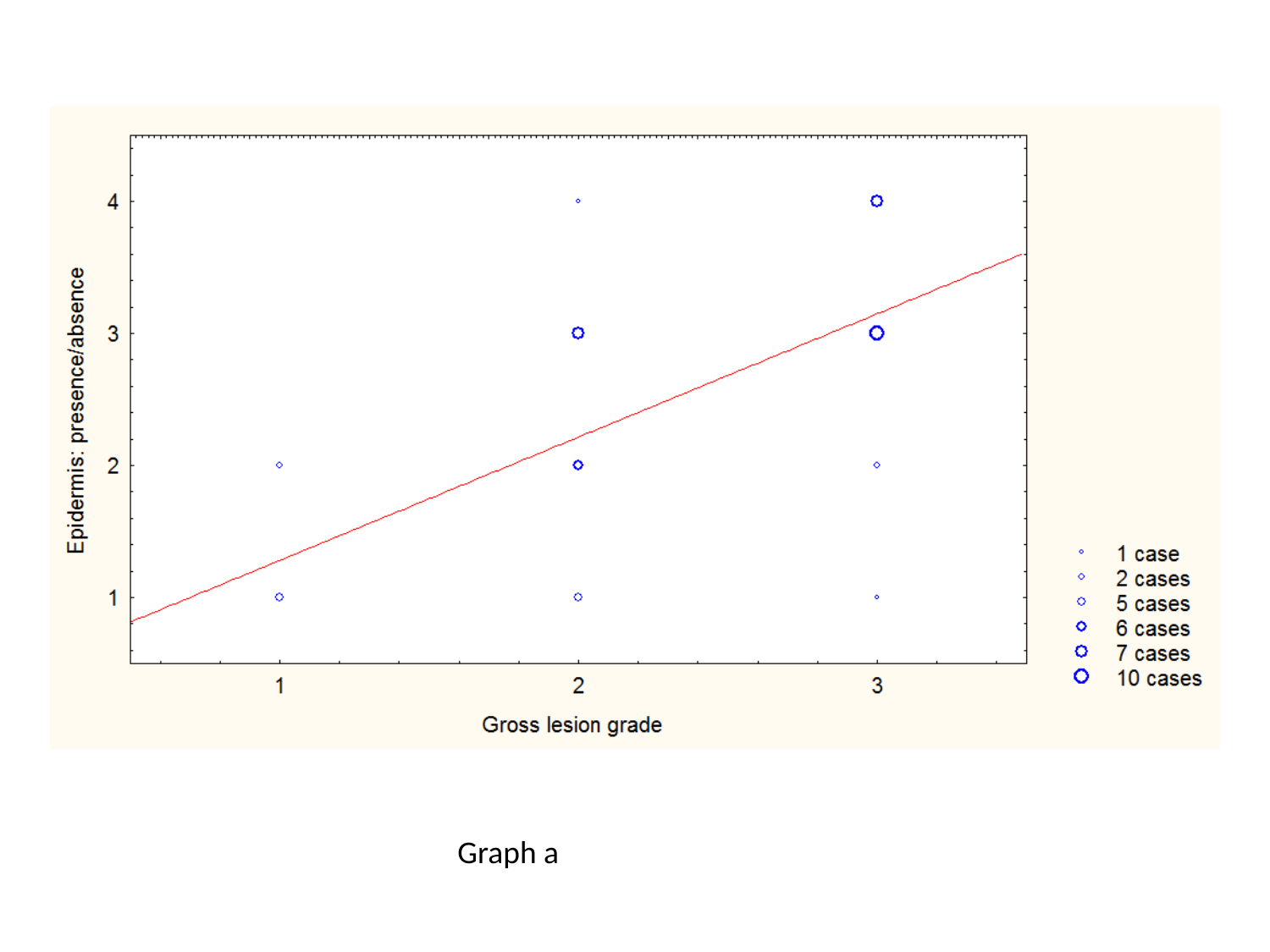

Graph a

## Slide 2
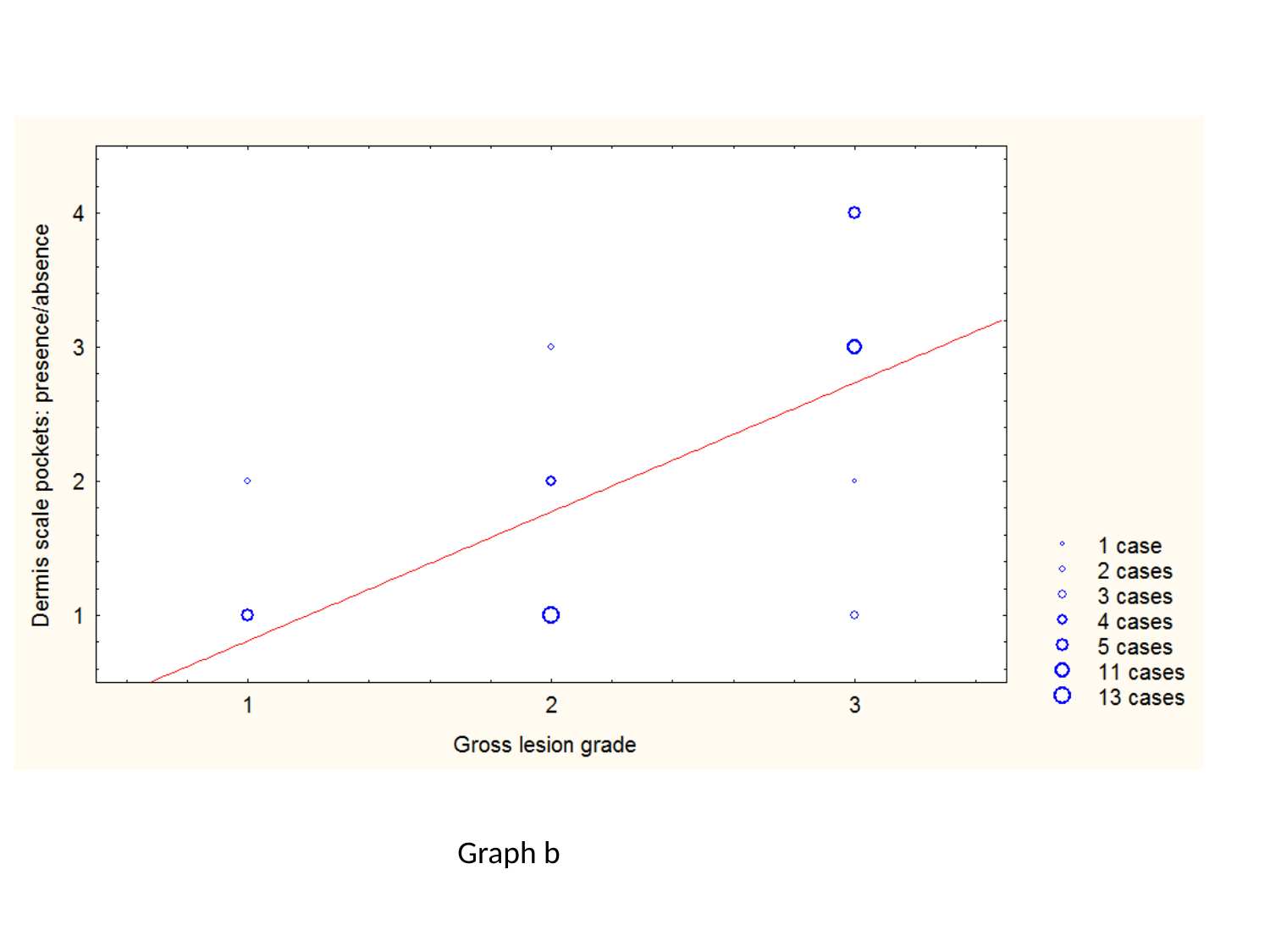

Graph b

## Slide 3
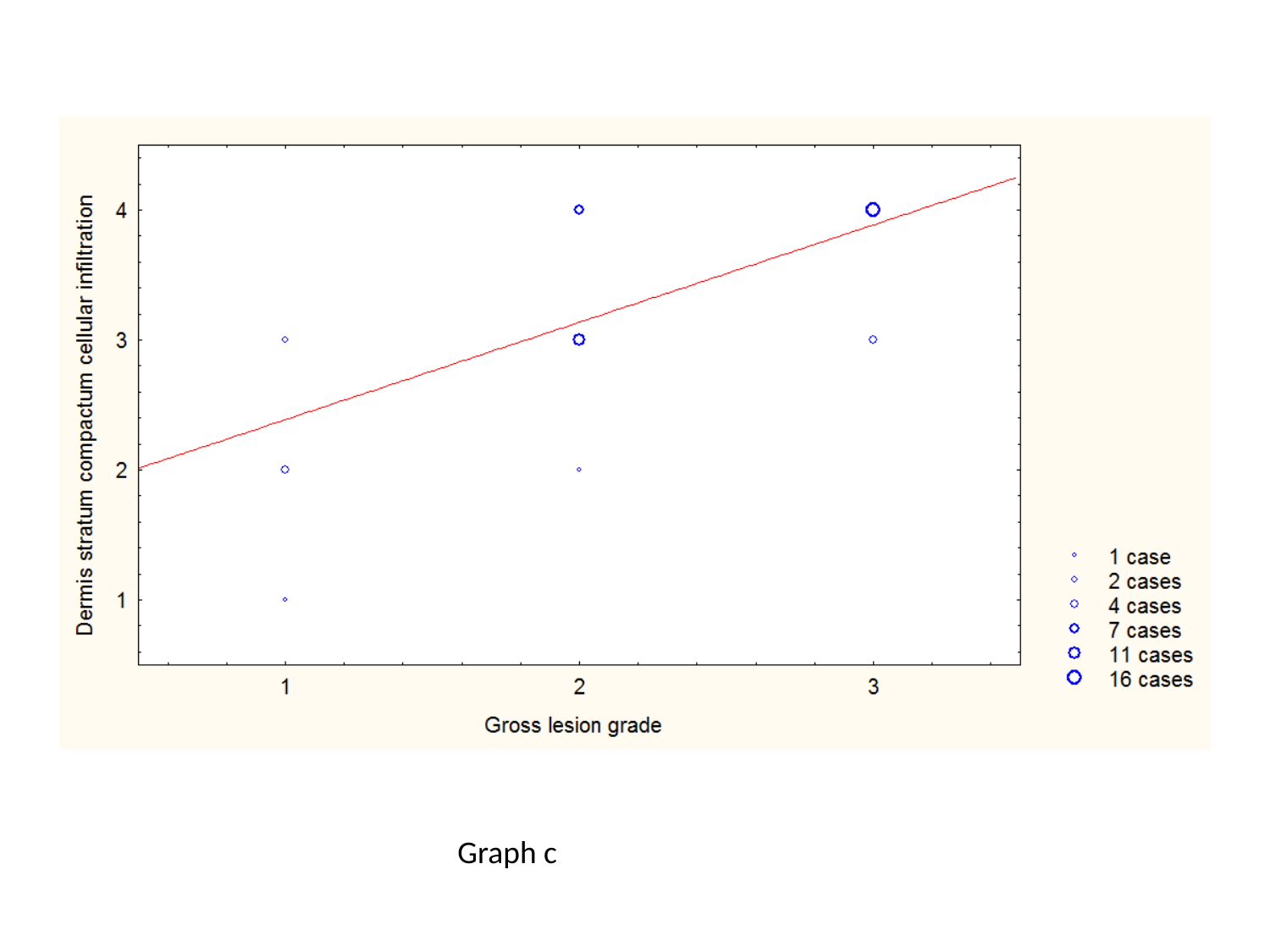

Graph c

## Slide 4
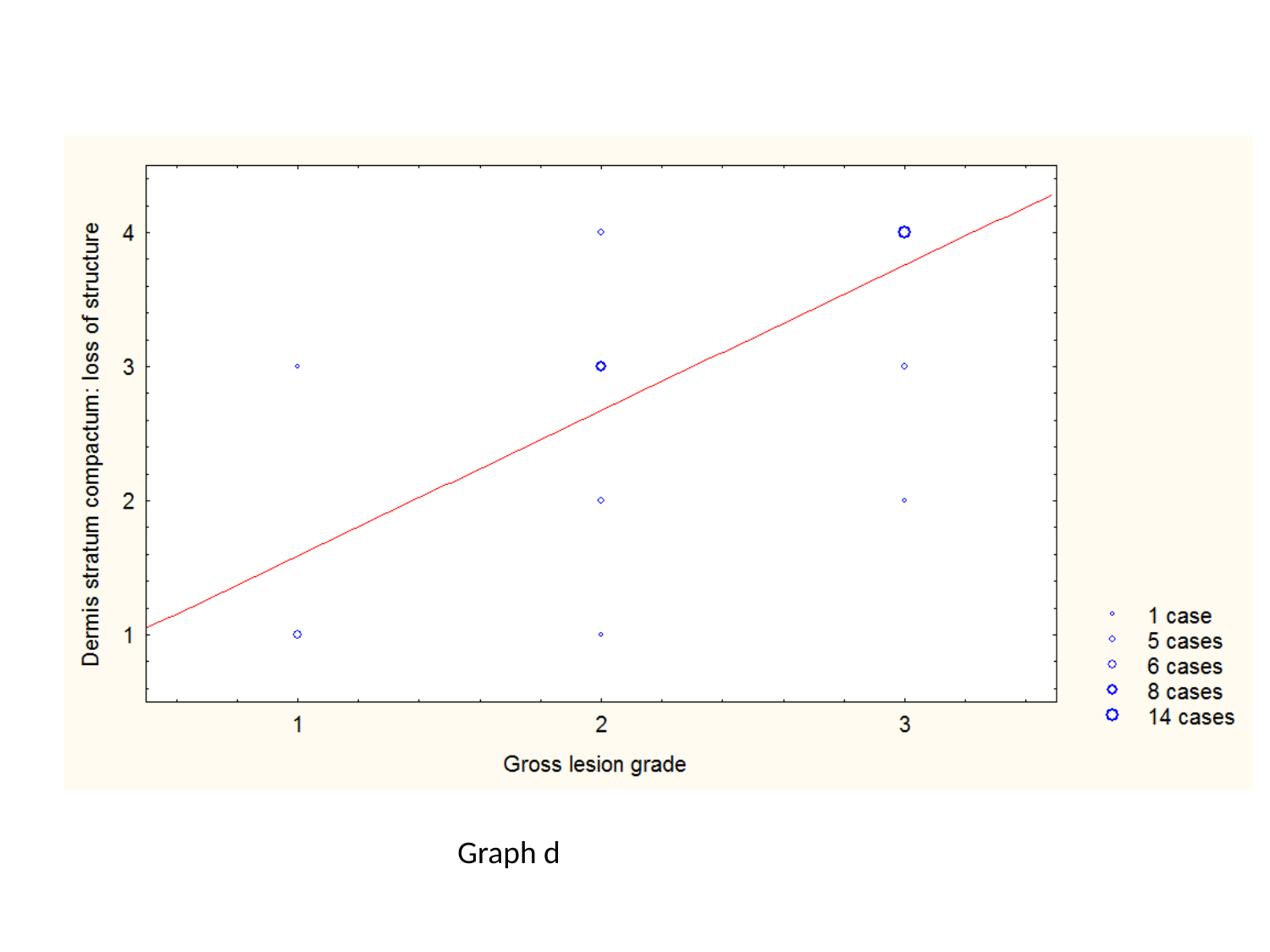

Graph d

## Slide 5
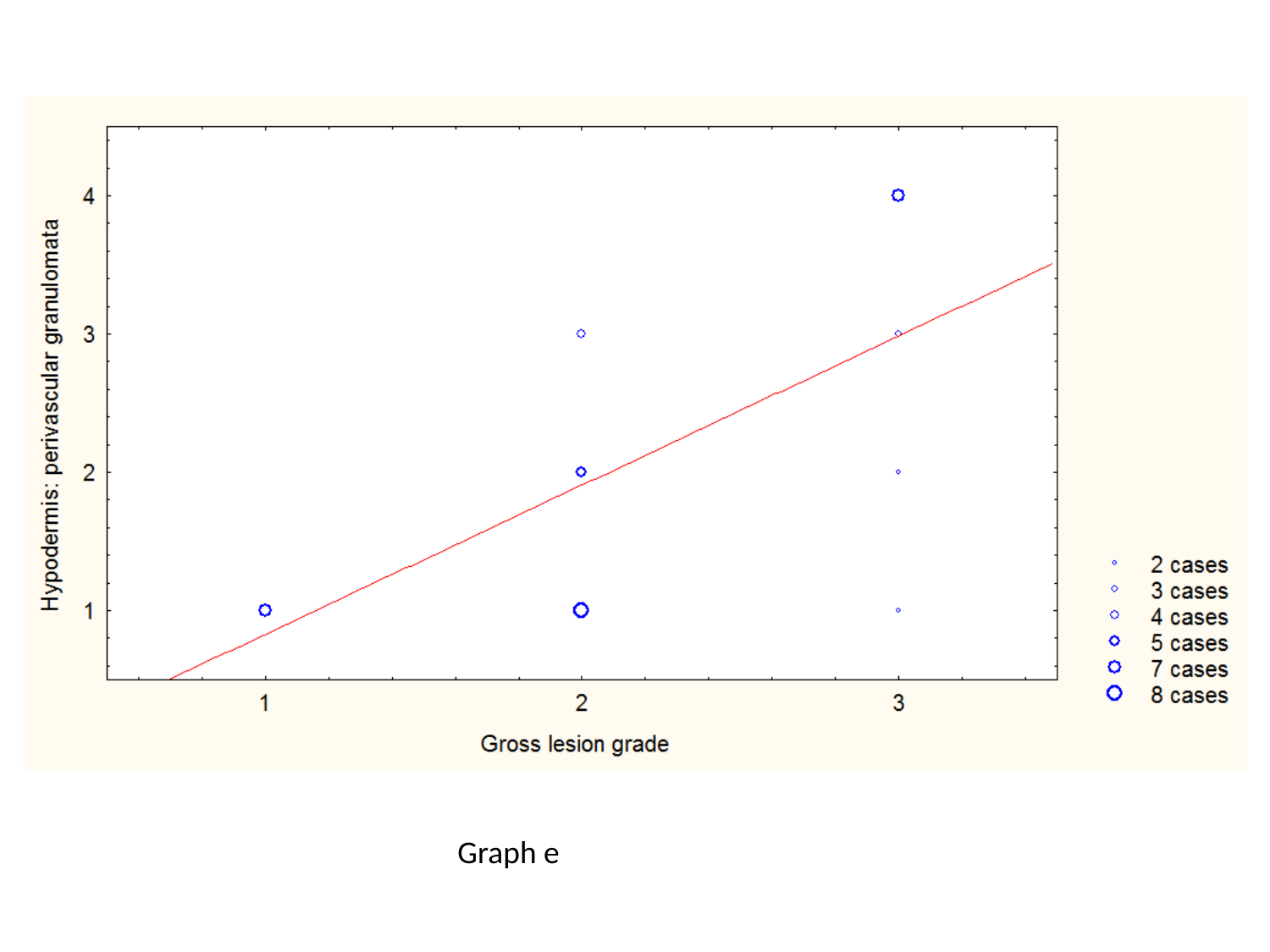

Graph e

## Slide 6
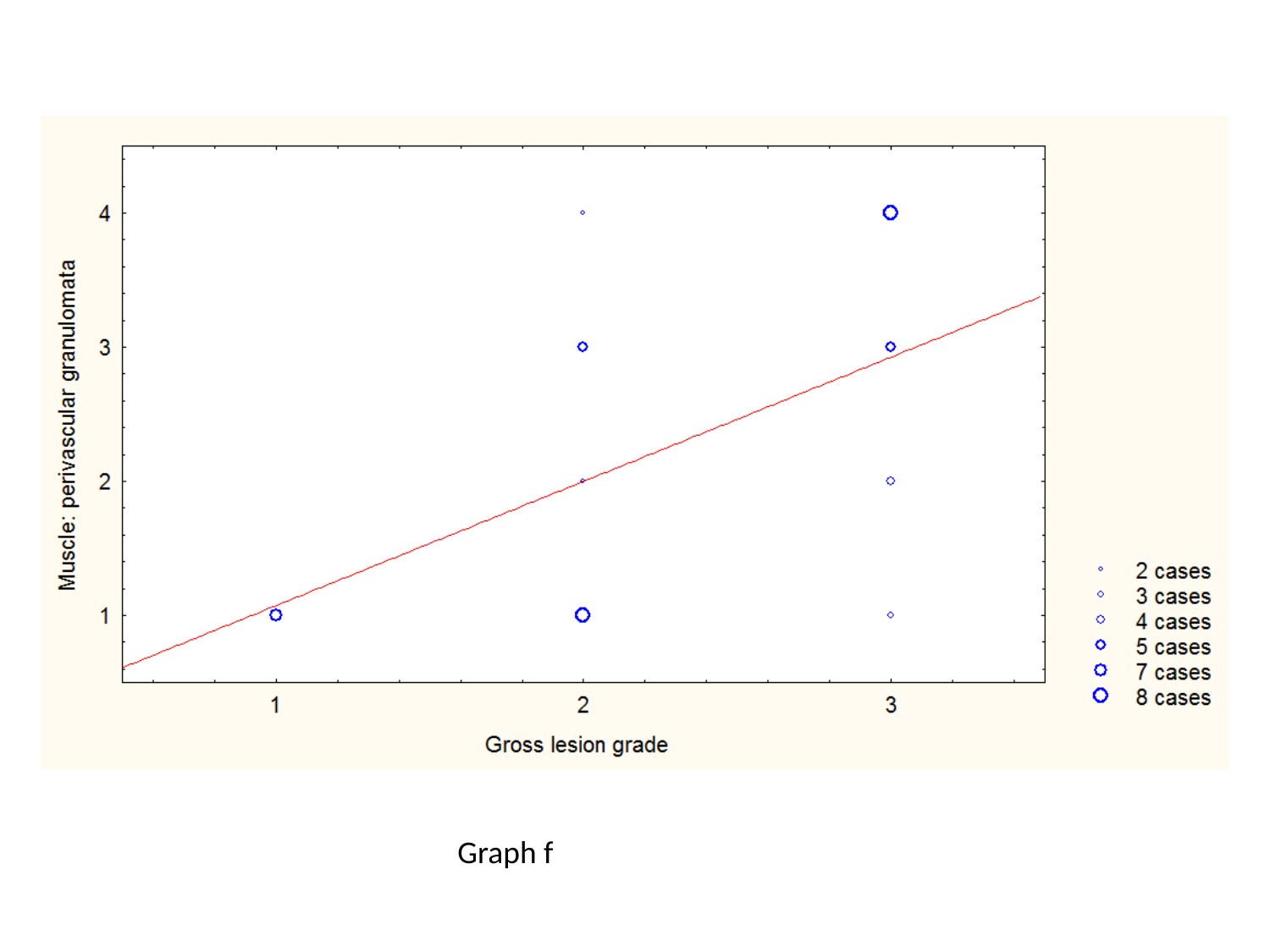

Graph f

## Slide 7
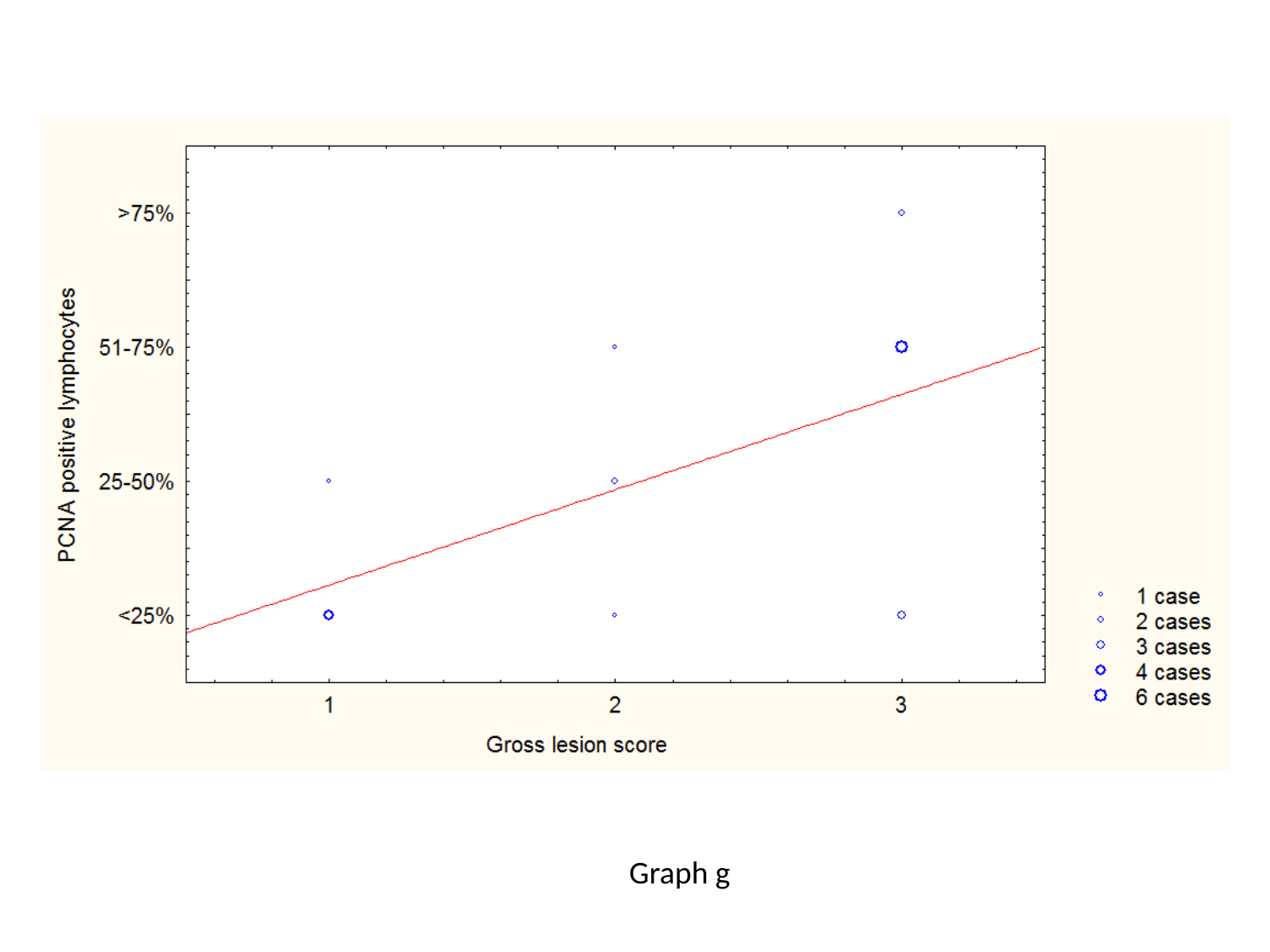

Graph g

Supplement: Supplementary file 1 — Supplementary Material [file JFD-44-1325-s001.pptx]
